# Immunological Aspect on Late Allograft Dysfunction

**DOI:** 10.1155/2014/625031

**Published:** 2014-04-14

**Authors:** Qiquan Sun, Akinlolu O. Ojo, Xian C. Li

**Affiliations:** ^1^Department of Renal Transplantation, The Third Affiliated Hospital, Sun Yat-sen University, 2693 Kaichuang Avenue, Luogang District, Guangzhou 510530, China; ^2^Department of Internal Medicine, University of Michigan, Ann Arbor, MI 48109, USA; ^3^Immunobiology and Transplantation Research Center, Houston Methodist Hospital and Texas Medical Center, Houston, TX 77030, USA

Organ transplantation is a well-accepted treatment for patients with end-stage organ failure. Despite continued improvement in short-term graft survival, late allograft dysfunction remains a significant problem in the clinic, especially in kidney transplant patients. Many factors contribute to late graft dysfunction, and among them immunological factors are the leading cause of late grafts loss. In this special issue, we have solicited a set of interesting papers addressing some aspects of this important topic in the field.

Here we focused on antibody-mediated rejection (AMR), as AMR is emerging as a major barrier to long-term graft survival, and the donor-specific antibodies are directly involved in AMR. The paper by Q. Sun and Y. Yang provides a comprehensive overview of the characteristics of AMR, both in acute rejection and late chronic rejection, and the current strategies in managing AMR. S. Lionaki et al. provide us with an excellent review on the incidence and clinical significance of de novo donor specific antibodies (DSA) after renal transplantation. It is well recognized that transplant glomerulopathy is a very special entity of late AMR, and Dr. W. Hanf from Australia discussed the effect of donor HLA antibodies on endothelium in transplant settings. These three review papers provide an extensive overview on antibody reactivity and its correlation with late graft injury.

On the mechanistic side, while C4d remains a marker of a humoral response, recent evidence indicates that, in renal allografts, microvascular injury in the presence of donor antibodies is indicative of antibody mediated graft injury. X. Li et al. confirmed that AMR is characterized with early capillary dilation, which is strongly correlated with intracapillary inflammation. They also discussed the diagnostic value of transcription factors T-bet/GATA3 ratio in predicting AMR, which may be of value in diagnosis of C4d-negative AMR.

The treatment of chronic ongoing AMR remains controversial. A group headed by C. W. Yang investigated the effect of combination therapy with rituximab and intravenous immunoglobulin on the progression of chronic AMR. They found that this therapy can delay the progression of chronic AMR. However, higher baseline proteinuria levels are associated with poor response to the treatment.

In another paper, H. Petra et al. evaluated differences in the intrarenal expression patterns of immune related genes in acute and chronic rejections. They found that Banff 2007 chronic rejection categories did not differ in intrarenal expression of 376 selected genes associated with immune response.

There are evidences that characters in early immune responses may also affect late graft function. L. Ma et al. reported that arterial lesions of donor kidneys had significant effects on the renal allograft function 2 years after transplantation, and correlations between donor age and arterial lesions were significant in this regard. Y. Wu and colleagues found that a novel tissue protective peptide, helix B surface peptide (HBSP) derived from erythropoietin, can effectively improve renal function by reducing tissue damage caused by ischemia reperfusion or cyclosporine A. The mechanism might be related to reduced caspase-3 activation, a key trigger in cell apoptosis and inflammation. Whether HBSP can display similar effect in clinical practice remains to be determined.

It should be noted that liver transplantation invokes relatively weak immunological responses. E. Muñoz-Sáez et al. reported the experimental design of an in utero hepatocellular transplantation model in rats, which is an interesting tool for investigating immune responses in other settings as well. Concerning clinical liver transplantation, data from S. Mizuno et al. suggested that peripheral blood CD4+ adenosine triphosphate activity (ATP) assay (ImmuKnow assay) can be useful in monitoring immunological aspects of transplant responses, while Y. Hu et al. documented that APACHE IV is superior to MELD scoring system in prognosis of transplant outcomes in patients with liver transplants, which may be used to improve the outcome of liver transplantation.

We anticipate that these outstanding papers will provide the readers with a comprehensive view on some of the challenging problems facing the transplant field. It is our hope that, by understanding the fundamental immune mechanisms in late allograft dysfunction, new and much improved strategies could be developed to further enhance transplant outcomes.

*Qiquan  Sun* *Qiquan  Sun* *Akinlolu O.  Ojo* *Akinlolu O.  Ojo* *Xian C.  Li* *Xian C.  Li*

